# Optimal feed profile for the Rhamnolipid kinetic models by using Tabu search: metabolic view point

**DOI:** 10.1186/s13568-016-0279-8

**Published:** 2016-11-16

**Authors:** J. Satya Eswari, Kannekanti kavya

**Affiliations:** Department of Biotechnology, National Institute of Technology Raipur, Raipur, 492010 India

**Keywords:** Tabu search, Rhamnolipid, Kinetic constants, Optimal control profile, Genetic algorithms

## Abstract

Rhamnolipids are bio surfactants which are extra-cellular glycolipids composed of l-rhamnose and 3-hydroxyalkanoics. Rhamnolipids are produced through fermentation process by using *Pseudomonas* sp. as the species. An alteration to the traditional procedures in order to achieve increase in the production of biosurfactants, a numerous process technologies have been adopted in fed batch mode. Fed batch mode facilitates the high production of product by avoiding the substrate or product inhibition line of attack. To overcome the controlling parameters which reduce the product yield, optimal control profiles are designed. In order to develop viable control methods for fed-batch fermentation of Rhamnolipid production, multiple substrate feeding strategies were employed and their efficiencies were compared with different substrates concentration of glucose, nitrogen and phosphorous. The product formation depends upon the substrate feeding strategy and so, the fed-batch fermentation was carried out by using *P. aeruginosa* providing substrates at manifold rates. With the obtained experimental data, using the kinetic models (logistic equation and by Luedeking Piret), the kinetic parameters were estimated. These kinetic parameters were implemented in tabu search algorithm and this programme was executed in Dev-C++, optimal control profiles were generated as a result. These obtained optimal control profiles have shown an increase in productivity of rhamnolipid with a decline in computational time. Through this procedure, the optimal control profiles of substrate feeding strategies of glucose, nitrogen and phosphorous were estimated. In comparison with other algorithms like genetic algorithm, Tabu Search algorithm was able to generate an accurate optimal control profiles with a reduction in their intricacy.

## Introduction

Rhamnolipids are vital surfactants which are studied under glycolipids. These are surface-active metabolites produced by *Pseudomonas* sp. They are widely studied under glycolipid biosurfactants that possess the ability to reduce surface tension of water as the standard value reduces from 72 to 30 mN/m, and the interfacial tension of water/oil systems reduces from 43 mN/m to standards of about 1 mN/m (Satya Eswari et al. 2013, 2016; Satya Eswari and Venkateswarlu 2016a, b; Prabu et al. 2015). These are produced by using a number of substrates such as glucose, pyruvate, glycerol, succinate, molasses, vegetable oils, starch-rich waste from potato processing, hydrocarbons, waste fruit processing, waste crop residue and agro industrial waste etc. Rhamnolipids are used as a source for rhamnose, for the production of high-quality flavor compounds which have a number of applications in the cosmetic and healthcare industries and in the biodegradation and bioremediation of xenobiotic controls. Inspite of their potential applications, Rhamnolipid could not compete with chemical surfactants due to their production budget and restricted productivity by microorganisms. This issue can be overcome with the use of higher yielding strain controlled systems, with metabolic engineering techniques, by adopting strain improvement methods, with low capital and operating cost processes, by use of media with economical substrates, by optimizing process parameters, and culturing strategies with minimal or manageable by-products. Fermentation has three modes of operation namely the batch, the fed-batch and the continuous process modes. Rhamnolipid Assembly by *Pseudomonas aeruginosa* in batch and fed batch routes have been studied and reported (Noh et al. 2014; Zhu et al. 2012). Batch process occasionally leads to substrate inhibition and catabolite repression responsible for reducing the productivity of Rhamnolipid. Fed batch mode facilitates the high production of products by avoiding the substrate or product inhibition strategies. The fed batch process of Rhamnolipid production is established to study and find the effect of substrate concentrations such as glucose, nitrogen and phosphorous. This paper represents fed-batch process with the mode of constant and exponential feeding control profiles are established to study the effects of glucose, nitrogen and phosphorous substrates to acquire the determined Rhamnolipid formation from *P. aeruginosa*. This paper also presents a study of the influence of various bioprocess parameters and optimization of control parameters. Kinetics of Rhamnolipid construction conditions were studied from (Delima et al. 2009). Development of control profiles by using various methodologies such as Tabu search and genetic algorithms were explored in bio and chemical processes due to their enormous advantages. (Beluham et al. 1995; Chen et al. 1998; Lin and Miller 2004). The main objective of this study is to build a mathematical model that relies on its potential parameters to estimate the quantity of the Rhamnolipid production by *P. aeruginosa.* These estimated parameters are introduced in biomass production. The substrate and product kinetic models for Rhamnolipid production are solved by using mathematical models and the Tabu search algorithm is used to develop the optimal control profiles. The generation of these control profiles plays a crucial role in overcoming the limitations of microbial surfactants for commercial utilization.

## Materials and methods

In this fed batch experiment, the *P. aeruginosa* strain was collected and cultured in 250 ml MCKeen Media, by maintaining pH 7.0 at 30 °C. The limiting substrates were in portion of 250 ml of MCKeen media with a composition of glucose (10.0 g/l), NH_4_NO_3_ (1.7 g/l), yeast extract (5.0 g/l), MgSO_4_·7H_2_O (0.2 g/l), KH_2_PO_4_ (3.0 g/l) and Na_2_HPO_4_ (7.0 g/l). This complete procedure is carried out in an orbital shaking incubator at 160 rpm. After 48 h of incubation the samples are collected and centrifuged at 8000 rpm for 10 min and supernatant is collected. By using two feeding strategies Limiting substrates were added to MCKeen Media at particular time intervals, namely: Constant Feeding Strategy and Exponential Feeding followed by Constant feeding strategy.

### Kinetic modeling and kinetic parameter estimation

#### Batch and fed batch studies kinetic parameter estimation and kinetic model construction

These models express that growth and product formation as a function of only biomass with evolution over time. Changes in biomass and Rhamnolipid production were represented by equations. Temporal variations in nutrient levels (glucose, nitrogen, and phosphorous) were defined by equations at Table 1.

**Table 1:** Batch and fed batch kinetic equations

$\frac{dx}{dt} = \mu_{m} \left[ {1 - \frac{x}{{x_{m} }}} \right]x$	Equation (1)
$x = \frac{{x_{m} x_{0} e^{{\mu_{m} t}} }}{{x_{m} - x_{0} + x_{0} e^{{\mu_{m} t}} }}$	Equation (2)
$p = \alpha \frac{{x_{m} x_{0} e^{{\mu_{m} t}} }}{{x_{m} - x_{0} + x_{0} e^{{\mu_{m} t}} }} + \beta \frac{{x_{0} }}{{x_{m} }}\ln \left( {1 - \left( {\frac{{x_{0} }}{{x_{m} }}} \right)} \right)\left( {1 - e^{{\mu_{m} t}} } \right)$	Equation (3)
$p = \alpha A(t) + \beta B(t)$	Equation (4)
$s = \gamma \frac{{x_{m} x_{0} e^{{\mu_{m} t}} }}{{x_{m} - x_{0} + x_{m} x_{0} e^{{\mu_{m} t}} }} - \eta \frac{{x_{0} }}{{\mu_{0} }}\ln \left( {1 - \left( {\frac{{x_{0} }}{{x_{m} }}} \right)} \right)\left( {1 - e^{{\mu_{m} t}} } \right)$	Equation (5)
$s = \gamma A(t) - \eta B(t)$	Equation (6)
$A(t) = \frac{{x_{m} x_{0} e^{\mu }_{m} t}}{{x_{m} - x_{0} + x_{m} x_{0} e^{\mu }_{m} t}}$	Equation (7)
$B(t) = \frac{{x_{0} }}{{\mu_{0} }}\frac{{x_{0} }}{{x_{m} }}\ln \left( {1 - \left( {\frac{{x_{0} }}{{x_{m} }}} \right)} \right)\left( {1 - e^{{\mu_{m} t}} } \right)$	Equation (8)
$q_{{s_{1} }} = \frac{1}{{Y{x \mathord{\left/ {\vphantom {x s}} \right. \kern-0pt} s}}}U_{s} + M$	Equation (9)
$q_{{s_{2} }} = \frac{1}{{Y{x \mathord{\left/ {\vphantom {x s}} \right. \kern-0pt} s}}}U_{s}$	Equation (10)
$q_{{s_{3} }} = \frac{1}{{Y{x \mathord{\left/ {\vphantom {x s}} \right. \kern-0pt} s}}}U_{s}$	Equation (11)
$q_{{p_{{}} }} = \frac{1}{{Y{p \mathord{\left/ {\vphantom {p s}} \right. \kern-0pt} s}}}U_{s}$	Equation (12)

#### Cellular growth modelling

The specific growth rate was expressed as a function of cellular growth only by means of the logistic equation described by Verhulst (1845) and Pearl and Reed (1920) in Bailey and Olli’s where μ_max_ is the maximum specific growth rate (1/h) and X is the maximum obtained cell concentration is shown in Eq. (1) in Table 1.

#### Product formation

Product formation kinetics described by Leudeking Piret model. In this work, model used to predict the Rhamnolipid concentration during the time passage of fermentation. The Leudeking Piret model combines both growth associated and non-growth associated contributions. Rendering to this model, the product construction level depends upon both the instantaneous biomass concentration, X and cell progression rate, dx/dt in a direct linear style. Where *α* and *β* were growth and non-growth associated constants that may vary with fermentation conditions. The graph was plotted between [P] vs. (t) gives the constant α and *β*. Shown in Table 1, Eq. (3) supported by Eq. (4).

### Substrate consumption and yield coefficients

A part of the substrate used for conversion of cell mass, a part used for product formation and another part for maintenance Table 1, Eq. (5) supported by Eq. (6). Yield coefficients of substrate and yield coefficients of product. The ratio of amount of biomass produced to the amount of substrate utilized is sited at Table 1, Eqs. (9, 10, 11 and 12) represents the ratio of amount of biomass produced to amount of substrate utilized in Fed-Batch Fermentation.

### Theory and optimization algorithm

#### Tabu search algorithm

This paper presents the solicitations of the artificial intelligence method called Tabu Search to design the feed rate controller of a bioprocess. The Tabu Search model is suitable for the work presented in the paper as the reiterative calculation is needed for searching the best solution for the control profile. The results show that this technique provides the better output waveforms compared with those designed from the classical method.

#### Tabu search engine—optimal control

**Fig. 1:**
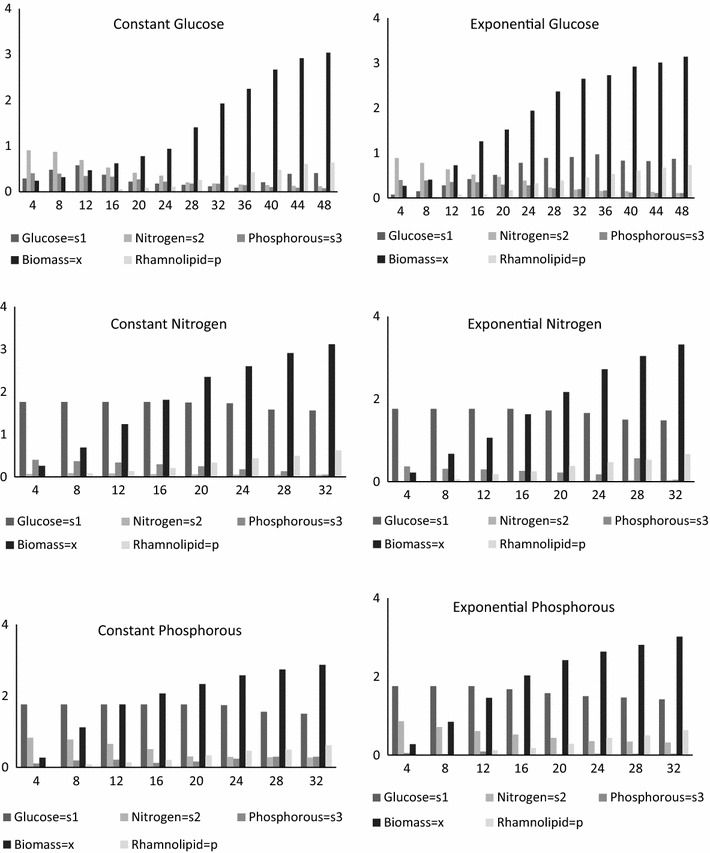
X-axis represents time interval, Y-axis represents substrates (S1,S2,S3) and rhamnolipid (P)

The Tabu search algorithm is search engine which is required to find the global optimum of a function over solution space of all possible solutions. Tabu Search uses particular coordinated strategies using adaptive memory to generate progressive sequence solutions. These progressive sequence solutions are then recognized as the best solutions, which are picked for recurring iterations. The current best solutions are stored in distinctive memory. The Tabu list is familiarized in this way in order to escape the possibility of selecting previously visited solutions. Another benefit of this memory usage in Tabu list is that it helps to escape local optimal solutions and to prevent cycling, and the information in Tabu list helps to guide the move from the current solution to the next solution. The fundamental version of Tabu Search used in this study is depicted in the schematic flowchart in Fig. 1.

The first step in Tabu Search is involved in the generation of initial solutions randomly. The second step is the neighborhood generation for each of these initial solutions. The current solution collectively moves with neighborhood of impending “next solutions” with the shortest path. An aspiration criterion allows the Tabu search algorithm to prevent any occasional moves which lead to unvisited solutions. The aspiration criterion is a condition under which the Tabu status of a certain move can be superseded. After reaching optimum path, the Tabu list is prepared. When best neighbor is not superior to the current solution, it is categorized as a Tabu and added to the recency-based Tabu list. As new solutions are added to the Tabu list, older solutions are unrestricted from the bottom. Thus, the Tabu list stores the most recently visited solutions and stops re-examining dubious solutions for a predefined number of iterations. It follows a restart mechanism as well, making decisions for future searches based on advanced information. The use of an adaptive reminiscence allows Tabu Search to “acquire” and generate a more flexible and effective search strategy than the “memory less” methods, such as simulated annealing (SA) and genetic algorithm (GA). Subsequently the neighborhood’s sieve to exclude Tabu moves, and a subset of candidate moves have been selected, and both the neighbor’s in the subset are evaluated. The best neighbor (having the highest evaluation) is selected, and becomes the “initial” solution for the next iteration. Often the objective functions contribute the base for the assessment, even though additional concerns can also enter. The objective function value itself can be manipulated before the selection. A stopping criterion is needed to stop the search process when the optimum is reached. The simplest form of stopping criterion could be to set a fixed number of iterations or a given computational effort.

### Genetic algorithms

Genetic algorithms are search based algorithms which have higher probability of finding a global optimum as they use potential solutions and probabilistic transition rules to create a set of new solutions. Genetic algorithms scramble the candidate solutions of the optimized algorithm as a string of characters which are usually binary digits. Genetic algorithms consider random strings to form inhabitants and updates iteratively in search of good solutions, which consider each iteration as a generation. A typical genetic algorithm requires a genetic representation of the solution domain and a fitness function to evaluate the solution domain. Once the genetic representation and the fitness function are defined, a GA proceeds to initialize a population of solutions and then to improve it through repetitive application of the mutation, crossover, inversion and selection operators. In order to attain the accuracy with a four bit coding, approximately 1/16th of the search space is needed. If the string length is increased by one, the accuracy increases exponentially to 1/32th of the search space. Taking the initial population size of an algorithm as N it considers the random strings as a variable Then compute the probability of each string derivative into the reproducing pool, by dividing with population size N. A fitness function F is derived and used in successive genetic operations Fang et al. (2003). The fitness of each discrete individual in the population is assessed through the cost function and new-fangled individuals are generated by using genetic operators (Changyu et al. 2007; Gupta and Sexton 1999). The genetic operators are involved in creating a list of solutions which perform various operations. In the crossover operation, new strings are produced by swapping information among strings in the reproducing pool. In single point cross over, two strings in the reproducing pool are selected at random and some portions of the strings are exchanged between the strings. The reproduction operator chooses noble strings and the crossover operator recombines noble strings together to optimistically create an improved sub-string (Varnamkhasti et al. 2012; Ozcelik and Erzulumla 2006). The mutation operator varies a string locally to optimistically create a novel string. In every cycle, new population is generated, estimated and verified for termination. The generational process is repeated until a termination condition has been reached. Common terminating conditions are said to be met when the solution is found that satisfies minimum criteria, when a fixed number of generations have been reached, or when allocated budget (computation time/money) has been reached. Other conditions for termination could be when the highest ranking solution’s fitness is reaching or has reached a plateau such that successive iterations no longer produce better results or by manual. If the termination criterion is not met, the population is iteratively operated by the above three operators and estimated.

## Results

### Experimental results

**Fig. 2:**
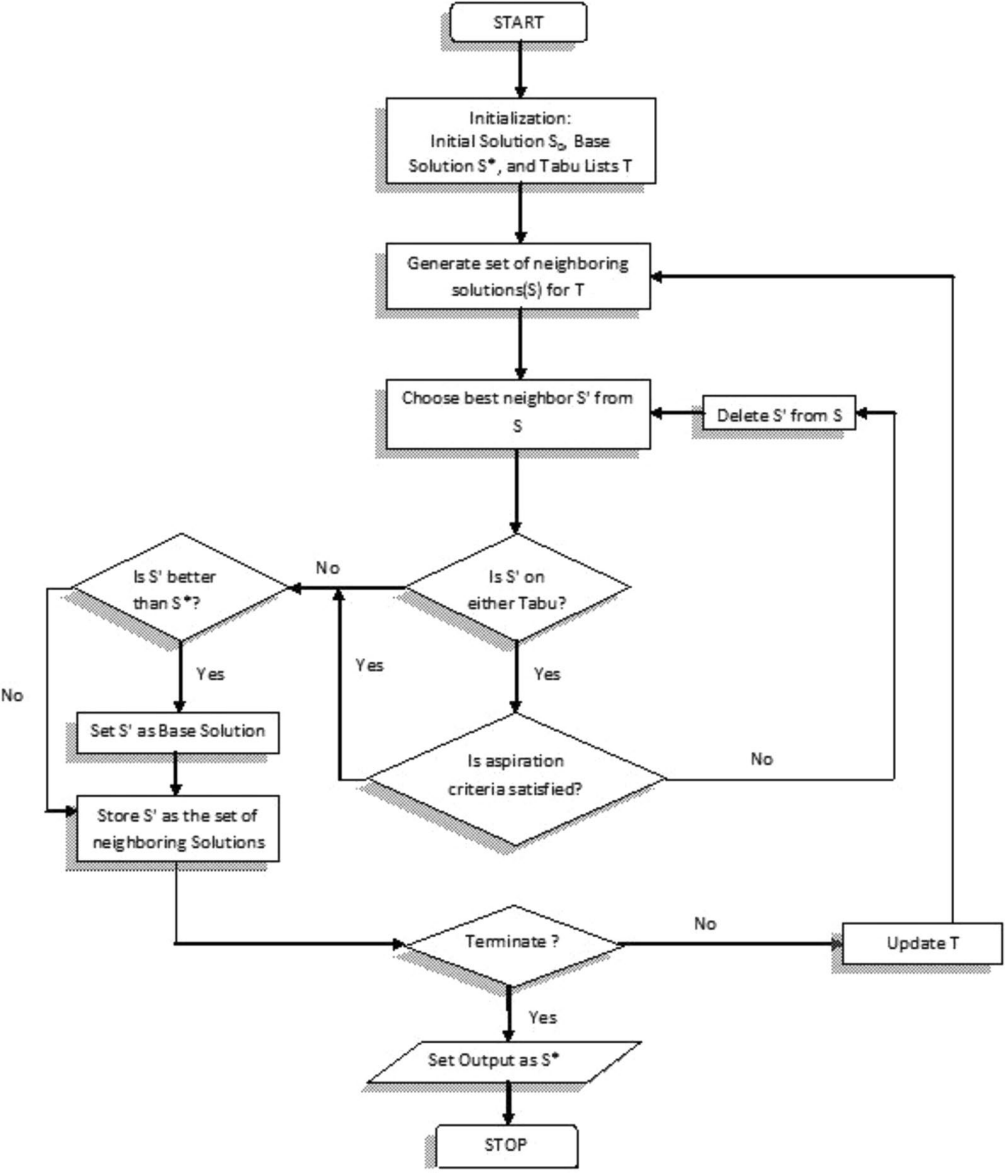
Tabu search algorithm

Experiments are conducted by using the fed batch mode for Rhamnolipid production by using *P. aeruginosa* with both constant and exponential substrate feeding strategies. With a time interval of 4 h the substrate feeding concentrations were noted down. After conducting experiments the Rhamnolipid, biomass and substrate depletion was measured. Experimental data is represented in Fig. 2.

### Batch kinetic parameter estimation results

The specific growth rate was expressed as a function of biomass only by using the logistic equation. Batch kinetic parameters are calculated with the help of logistic and Leudeking Piret Models. The obtained experimental data represented in Fig. 2 is then used to establish the batch kinetic constants. By using multiple linear regressions, the estimated batch kinetic constants for substrate depletion and product formation and given in Table 2 and estimated parameters are represented graphically in Fig. 1.

**Table 2:** Batch kinetic constants

	Alpha (*α*)	Beta (*β*)
Glucose	0.6111	0.0002
Nitrogen	0.0112	−0.000003
Phosphorous	0.1765	−0.000005
Rhamnolipid	0.3091	−0.000009

### Fed-batch kinetic parameter estimation

The development of a feeding strategy in a fed-batch culture for biomass growth to control the substrate concentration at its optimal level. This is essential to attain a maximal cell concentration and high biomass productivity. In addition, this approach affects the overall PHB (Poly-β-hydroxybutyrate) productivity by preventing the premature shifting to phase 2. In this study, two types of substrate feeding strategies were employed one is the constant feeding strategy where the other exponential substrate feeding strategy to maintain the substrate concentrations within an optimal range. First, a series of experiments were performed to determine the initial substrate concentrations (S0) such as glucose (G0), nitrogen (N0), and phosphorous (P0). These results indicate that the initial concentration of the substrate significantly affects the specific growth rate, which is found to be at a maximum at the initial substrate concentration. A decrease in growth rate was observed at the higher substrate concentrations. By using standard models such as the logistic model and Leudeking Piret Model, the kinetic parameters were estimated mention in Table 3 which represents all the kinetic constants determined by graphical regression method by constant and exponential feeding strategies using glucose, nitrogen, phosphorous as substrates.

**Table 3:** Fed batch kinetic constants and Tabu search parameters

	Constant feeding rate			Exponential feeding rate		
	Glucose	Nitrogen	Phosphorous	Glucose	Nitrogen	Phosphorous
_Umax_	0.0258	0.1283	0.01161	0.1366	0.123	0.12
l1	0.01	0.01	0.01	0.01	0.01	0.01
h1	0.01	0.01	0.01	0.01	0.01	0.01
F	0.45	0.45	0.45	0.45	0.45	0.45
Ki	0.01	0.01	0.01	0.01	0.01	0.01
Si	0.29	0.071	0.1104	0.07	0.009	0.0558
Sgf	0.41	1.56	1.5	0.87	1.48	1.42
Snf	0.1196	0.0495	0.2779	0.0109	0.0393	0.3184
Spf	0.0755	0.0567	0.2987	0.1099	0.0453	0.0326
Alpha (*α*)	0.824	0.633	0.966	0.07512	0.0078	0.891
Beta (*β*)	1	0.699	2.59	5.0972	0.4246	0.282
Gamma (*γ*)	0.85	−12.725	2.4	0.6763	13.298	−3.177
eta (*η*)	0.45	−7.5772	1.3	2.5618	9.9924	−0.999
Ks	0.387	5.0972	1.28	0.683	0.0106	−0.00468
Muf	0.005	0.0086	0.006	0.005	0.011	0.008
Y	0.421	0.421	0.421	0.421	0.421	0.421
Xf	3.04	3.12	2.87	3.14	3.32	3.02
Pf	0.637	0.626	0.734	0.734	0.666	0.634
Sf	0.41	0.0495	0.87	0.87	0.393	0.0326

#### Constant and exponential feeding strategy

**Fig. 3:**
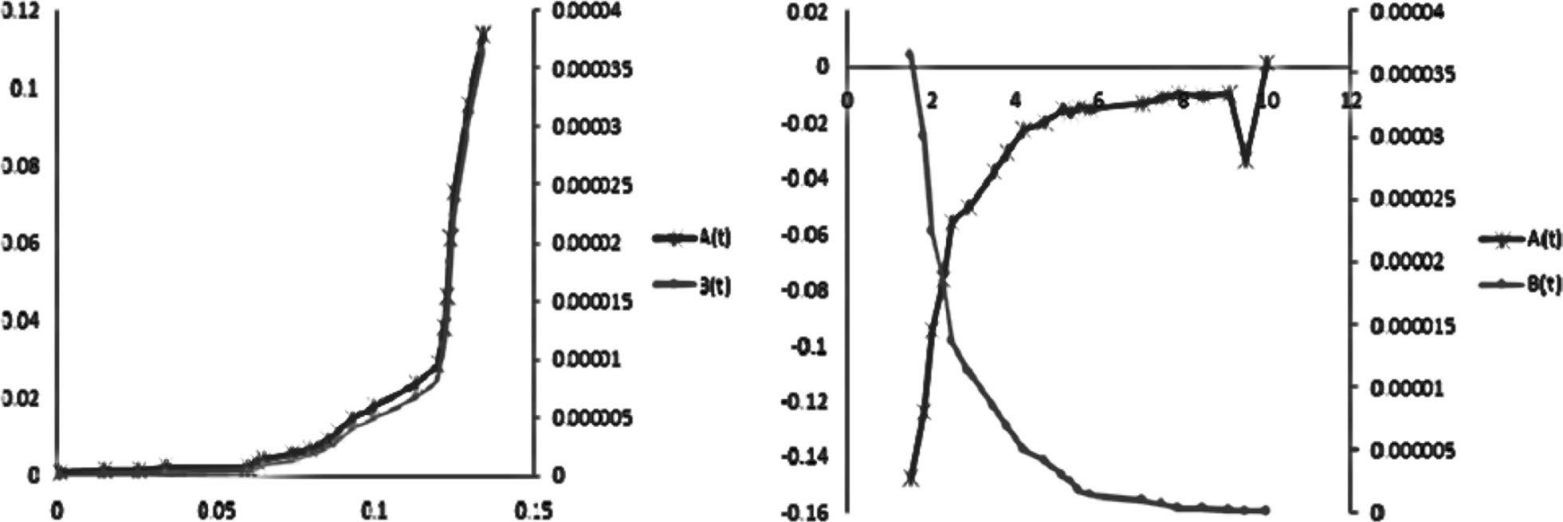
Batch kinetic constants and product constants graphically

**Fig. 4:**
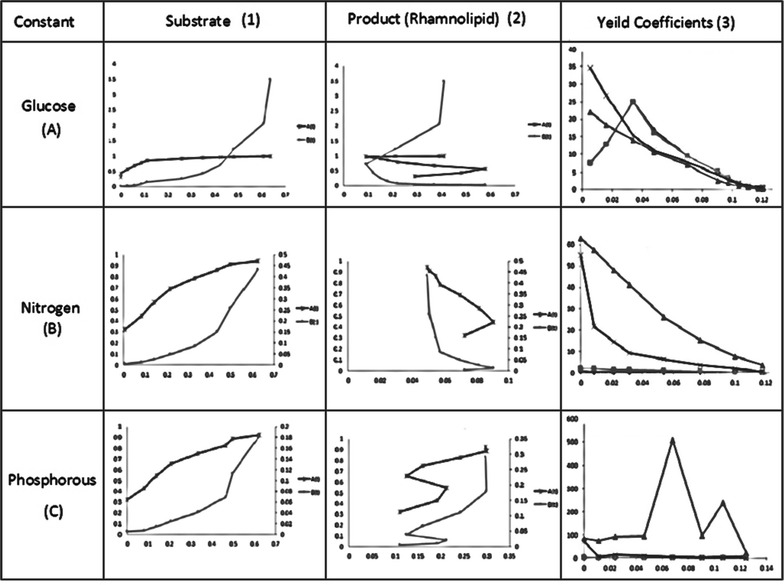
Constant feeding strategy with respect to time

During fed-batch fermentation, the glucose concentration has an optimal range for cell growth and synthesis of the target product. The growth of cell would be very slow if the glucose concentration fall below the lower limit, while the organic acids were synthesized to a great extent in the presence of high glucose concentration. Before establishing kinetic models for this process, the general range of glucose concentration must be determined. For this, Leudeking Piret kinetic models are established. By using the equations at Table 1 the kinetic models are solved with the experimental data. Table 4 represents constant substrate feeding strategy and exponential substrate feeding strategy parameter (1) signify constant substrate feeding strategy whereas parameter (2) signify exponential substrate feeding strategy both parameters consist of serial numbers which determine cell growth rate, product formation rate, substrate formation rate, yield coefficient of substrates, yield coefficient of product with glucose, nitrogen, phosphorous as substrates. In Fig. 3 the constant glucose, nitrogen, and phosphorous with the corresponding product are graphically represented, followed by exponential feeding strategy in Fig. 4.

**Table 4:** Kinetic model parameter substituted in equations

Parameters	Serial no.	Models	Glucose (A)	Nitrogen (B)	Phosphorous (C)
Constant feeding strategy (1)	1	Cell growth rate (*μ*_*m*_)	0.13	0.12830	0.1161
	2	Product formation rate (*α*, *β*)	0.824, 1	0.633, 0.699	0.966, 2.59
	3	Substrate formation rate (*γ*, *η*)	0.85, 0.45	−12.725, −7.5772	2.4, 1.3
	4	Yield coefficient of substrate (glucose)	−0.00948	−0.005	−0.00936
	5	Yield coefficient of substrate (nitrogen)	−0.06502	−0.00192	−0.0032
	6	Yield coefficient of substrate (phosphorous)	−0.05958	−0.00193	−0.003
	7	Yield coefficient of product	−0.00936	−0.30264	−0.25359
Exponential feeding strategy (2)	1	Cell growth rate (*μ*_*m*_)	0.1366	0.133	0.12
	2	Product formation rate (*α*, *β*)	0.07512, 5.0972	0.0078, 0.4241	0.891, 0.282
	3	Substrate formation rate (*γ*, *η*)	0.6743, 2.548	13.298, 9.9924	−3.177, −0.999
	4	Yield coefficient of substrate (glucose)	−0.48396	−0.00507	−0.00488
	5	Yield coefficient of substrate (nitrogen)	−0.06502	−0.00192	−0.0032
	6	Yield coefficient of substrate (phosphorous)	−0.05958	−0.00193	−0.003
	7	Yield coefficient of product	0.114277	−0.30264	−0.25359

### Tabu search optimal control strategy

**Fig. 5:**
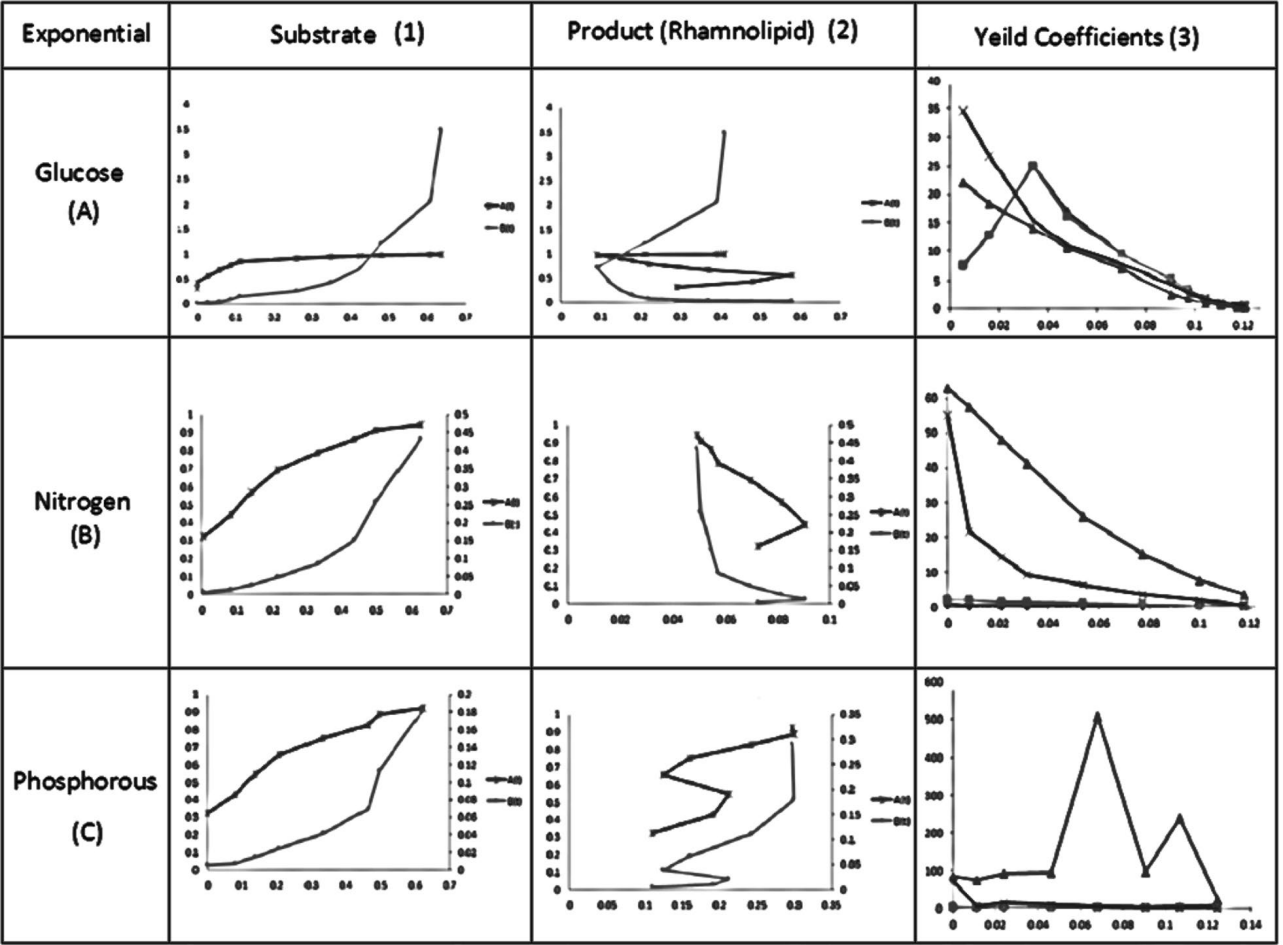
Exponential feeding strategy with respect to time

**Fig. 6:**
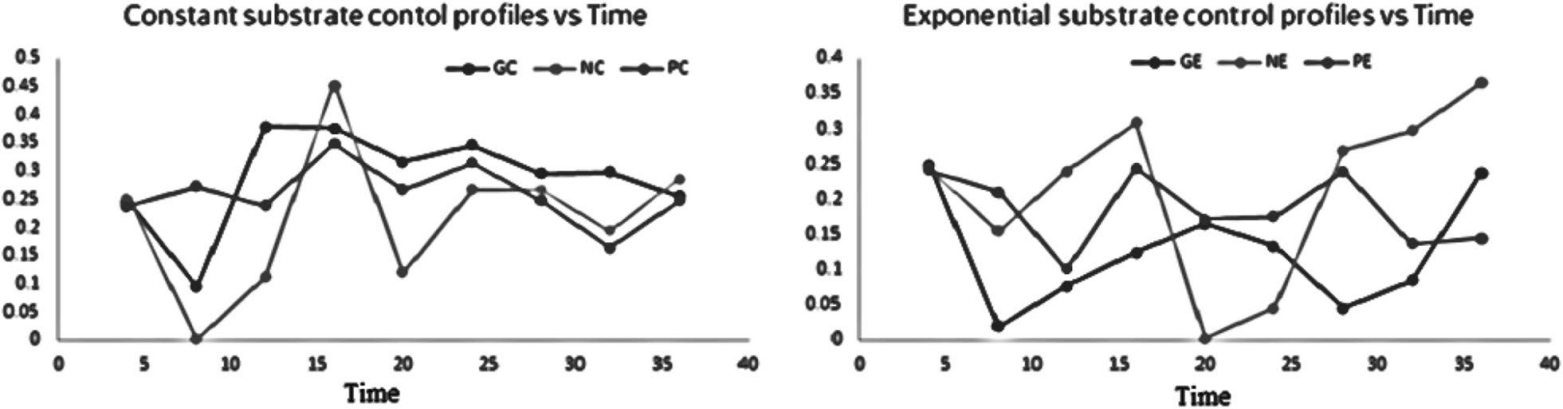
Optimal control profile for constant and exponential feeding strategy

The optimal control strategy of glucose, nitrogen and phosphorous were established by using Tabu search. The optimal parameters selected are given in Table 4. The algorithm is written as a program reference at Fig. 5 with a lower limit of −0.01 and with a upper limit of 0.01, with time interval as 4 h and final time given as 48 h followed by 5, 25, 50, 100 iterations initially which results in generations of new neighbor solutions. For the 5, 25, 50 iterations the solutions are formed by the respective equations and results into formation of Tabu list from these neighbor solutions best solution is picked and it is crosschecked whether it follows aspiration criteria or not the one which follows aspiration criteria that is the condition for convergence results into formation of base solution. These base solutions undergo termination which gives optimal output solution following each iteration. By tuning the C program with Lower limit as 0.01 and upper limit as 0.06 and by tuning various constant parameters such as setting iteration as 100, the glucose, nitrogen, phosphorus, both at constant and exponential feeding strategy give positive optimal solutions with a time interval of 4 h represented in Fig. 6.

### Comparative methods: genetic algorithms

The optimal control profiles (constant feeding and exponential feeding strategy with glucose, nitrogen and phosphorous) obtained by using genetic algorithms are generated. The optimal parameters selected are Pc = 0.7, P_m_ = 0.08, selection method is roulette wheel. With this method the Rhamnolipid concentration was not increased as expected.

## Discussion

**Fig. 7:**
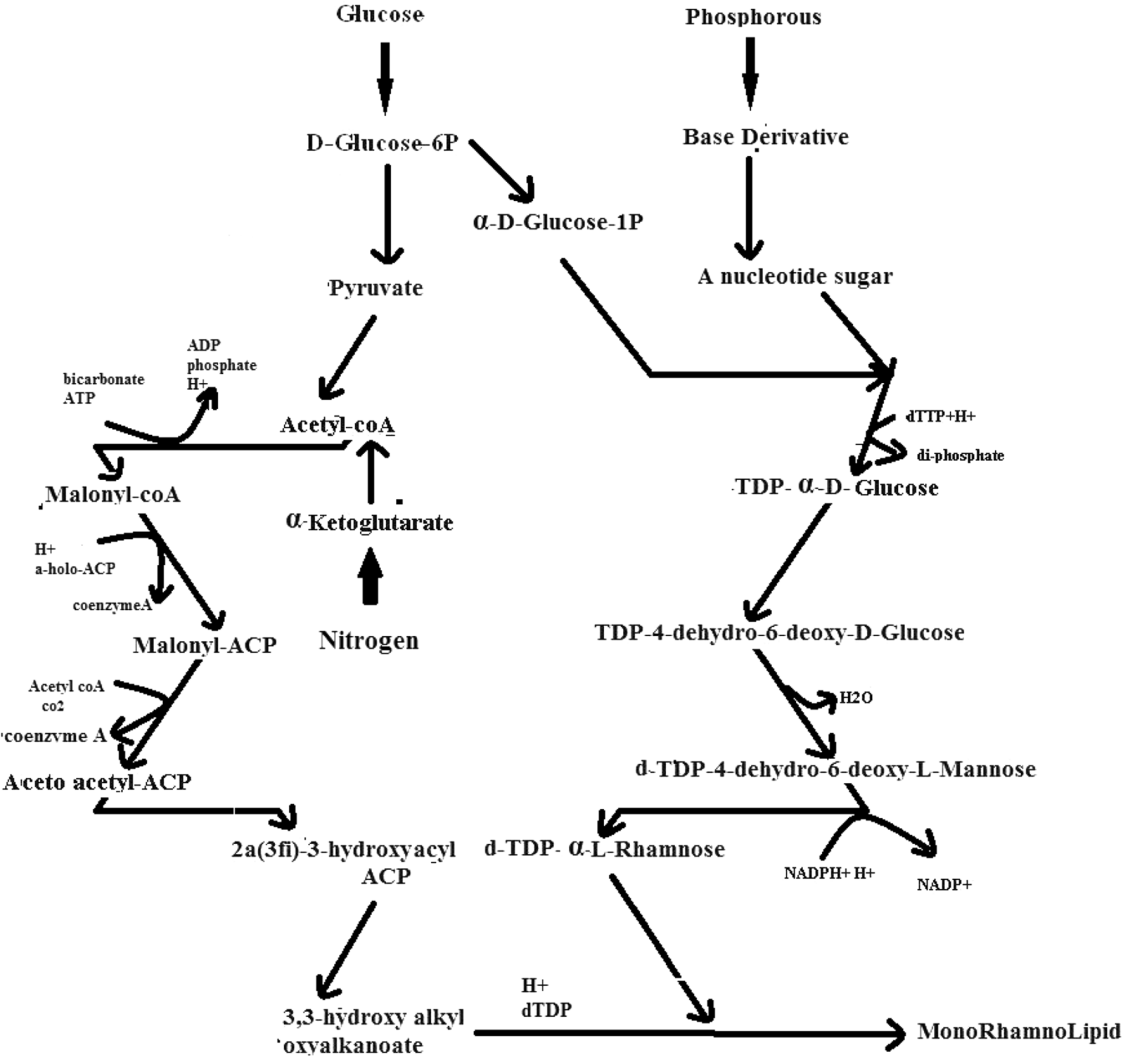
Rhamnolipid production from *Pseudomonas aeruginosa*

Nutritional requirements are major factors for Rhamnolipid enhancement process. The type, quality and quantity of bio-surfactant produced are influenced by the nature of the substrate, the concentration ions in the medium, and the culture conditions, such as pH, temperature, agitation and dilution rate in continuous culture (Rodrigues et al. 2007). The fed-batch processes start with the cells being grown under the batch conditions, usually until close to the end of the exponential growth phase. At this point, the solution of substrate (nutrients) is fed into the reactor, without the removal of the culture fluids. This feed should be balanced enough to keep the growth of the microorganisms at a desired specific growth rate and simultaneously reducing the production of by-products. Substrates such as glucose, nitrogen and phosphorous are added to the culture, and the metabolic changes were seen in the metabolic pathway of Rhamnolipid production by *P. aeruginosa*. Coming to the metabolic pathway of Rhamnolipid production by *P. aeruginosa* (Satya Eswari et al. 2016) which is given in Fig. 7, shows that the Rhamnolipid production is influenced by the presence of the three substrates depending on their feeding profiles. Glucose is converted as rhamnose with an intermediate supply of nitrogen to acetyl CoA and glucose is also involved to form a TDP-α-d-Glucose with intermediate supply of phosphorous. Three substrates such as glucose, nitrogen, phosphorous combination leads to mono Rhamnolipid production. Excess supply of these three substrates leads more amount of rhamnose production hence it may not be involved in lipid formation. Inadequate supply of these three substrates causes a condition of starvation and hence the produced rhamnose is consumed by the *P. aeruginosa.* In Fig. 6 optimal control profiles of both constant and exponential substrate feeding strategy are given. Represent the Importance of substrates and their feeding strategies in Rhamnolipid production.

Rhamnolipid as biosurfactants has got potential applications in medical, pharmaceutical and food industries produced by species *P. aeruginosa.* To reduce the effect of parameters on production of Rhamnolipid, and to meet the desired objectives of product formation, the control profiles of substrate feeding strategy were developed. The optimal feed rate for Fed-Batch Fermentation process of Rhamnolipid production by pseudomonas *aeruginosa* has been developed in this study. The Tabu search algorithm is applied to the fermentation process with multiple types of substrate feed strategies. Limiting the feeding strategies of substrates as a controlling parameter, the production of Rhamnolipid was increased. The various kinetic coefficients of constant and exponential feeding strategy are determined by using multiple linear regressions method. These limiting feeding strategies of substrates also act as a parameter for the Tabu algorithm. By using Tabu algorithm which was implemented in Dev-C++, the optimal control profiles were generated. These optimal control profiles reduce the singular control problems and the computational difficulties were not confronted. The results obtained were within the permissible limits when compared with the concentration of initial substrates (S_0_). The Tabu search algorithm may be very promising in obtaining practically feasible and desirable control profiles which is also important for challenging problems. Therefore, it is easy to use even for very complicated problems that regulate yield on singular control systems like substrates. In this study glucose, nitrogen and phosphorous was preferred for increasing Rhamnolipid production, and the glucose, nitrogen and phosphorous concentration feeding profiles were successfully generated. For comparative studies genetic algorithms are used and optimal control profiles are reported.

## Abbreviations

F: volumetric glucose feed rate (l/h); F_max_: maximum volumetric substrate feed rate (l/h); Ki: substrate inhibition constant for growth (g/l); K_0i_: substrate inhibition constant for Rhamnolipid production (g/l); K_s_: monod kinetic constant (g/l); K_s_^0^: saturation constant for q_p_ (h^±1^); P: ethanol concentration (g/l); p_m_: maximum Rhamnolipid concentration for cell growth (g/l); p_mP_: maximum Rhamnolipid concentration for Rhamnolipid production (g/l); p_i_: Rhamnolipid threshold concentration for Rhamnolipid production (g/l); q_p_: specific product formation rate (h^±1^); q_pm_: maximum specific Rhamnolipid production rate; S: substrate concentration (g/l); S0: initial substrate concentration (g/l); s_F_: feed concentration of substrate (g/l); Si: threshold substrate concentration for cell growth; V: working volume of the fermenter (l); V_F_: total working volume of the fermenter (l); x: biomass concentration (dry weight) (g/l); Xmax: maximum cell concentration at dense packing (g/l); Y_p/s_: Rhamnolipid yield (0.47 g/g); M: maintenance coefficient; U_max_: cell concentration growth rate (l/h); Sgf: Substrate glucose final value at time 48 h; Snf: substrate nitrogen final value at time 48 h; Spf: substrate phosphorous final value at time 48 h; Muf: maximum u value at specific feeding strategy; Xf: biomass value at time interval of 48 h; Pf: product value at time interval of 48 h; Sf: substrate value at time interval of 48 h at constant/exponential substrate feeding strategy; G0: initial glucose nitrogen phosphorous concentration (g/l); N0: initial nitrogen concentration (g/l); P0: initial phosphorous concentration (g/l); Alpha: growth associated parameter for product formation; Beta: non growth associated parameter for product formation; Gamma: growth associated parameter for substrate formation; Eta: non growth associated parameter for substrate formation.

## Tabu search algorithm

I: index of the neighbor solution; k: current iteration number; k_center_: determines at what fraction of total iterations s (k) = 0.5; L: length of both tabu lists; m: maximum number of iterations; n: coefficient determined according to the complexity of problem; N_Neigh_: total number of neighbor solutions generated at each iteration; N_var_: number of variables; P: random number with uniform distribution; S(k): value of the sigmoid function.
